# The Effect of COVID-19 on Length of Stay in Hospital and Patient Population Following Burn Injury

**DOI:** 10.1093/jbcr/iraf192

**Published:** 2025-10-06

**Authors:** Sara Sheikh-Oleslami, Bettina Papp, Anthony Papp

**Affiliations:** Division of Plastic Surgery, Department of Surgery, University of British Columbia, Vancouver, BC, Canada; Faculty of Science, Capilano University, North Vancouver, BC, Canada; Division of Plastic Surgery, Department of Surgery, University of British Columbia, Vancouver, BC, Canada

**Keywords:** length of stay, burns, pandemic, coronavirus, housing

## Abstract

Acute burn care is heavily resource-dependent and thus was significantly impacted by the COVID-19 pandemic. This study sought to examine the relationship between COVID-19 and the length of stay (LOS) in hospital following burn injury, as prolonged admissions have implications on both individuals and healthcare systems. Additionally, this study explored how COVID-19 affected the homeless burn population, as homelessness has been associated with longer hospital admissions due to limited post-discharge resources. Single-center, retrospective cohort study using data from the Burn Registry and medical chart review with inclusion of all adult burn patients admitted to a quaternary provincial burn unit from April 1, 2016, to March 31, 2023. Patients admitted prior to April 1, 2020, were considered the pre-COVID cohort. Key variables included demographic characteristics and LOS, with homelessness defined as a lack of a fixed address. Of 498 included patients, 301 and 197 were in the pre-COVID and COVID cohorts, respectively. While both cohorts had similar age and gender distributions, a significant difference was noted in LOS between cohorts, with COVID cohort patients staying in hospital for 22 (24) days compared to 20 (29) days in the pre-COVID cohort. More notably, a 58% increase in homeless patients was seen during COVID, with 17% (50/301) of admitted patients being homeless pre-COVID compared to 26% (52/197) during COVID (*P* < .05). The COVID-19 pandemic resulted in a slightly increased LOS in burn patients, with homeless patients disproportionately affected. This has important implications for both patient outcomes and healthcare resource allocation.

## INTRODUCTION

Burn injuries cause an estimated 180 000 deaths per year, with non-fatal burn injuries being a leading cause of morbidity.^1^^,^^2^ In 2023, over 398 000 burn injuries occurred in the United States of America, with approximately 30 000 requiring hospitalization.^3^ With devastating consequences and usually prolonged lengths of hospital stay, burns are also among the injuries with the highest costs of care, factoring in the cost of hospital stay, treatment, and subsequent rehabilitation.^4^

Many factors impact the length of stay (LOS) in the hospital following acute burn injury, including age, burn depth and extent (TBSA%), presence of inhalational injury, and incidence of infection.^4^ Advancements in burn management have greatly focused on the acute period following traumatic injury, with early treatment and rehabilitation greatly decreasing morbidity and mortality.^5-8^

These advancements were complicated with the onset of COVID-19, as many medical services, including burn care, were hindered by a lack of resources, personnel, and supplies.^9^ These disparities further grew with increased focus shifted toward managing COVID-19 patients.^10^ Many hospitals were unable to maintain reserves of caregivers, equipment, or supplies for other care services. Furthermore, outpatient services for patients following discharge from hospital, including wound care and rehabilitation, were less accessible due to closures of such facilities and COVID-related stay-at-home orders.^5^

A previous study exploring the impact of the COVID-19 pandemic on the incidence and epidemiology of adult kitchen-related burn injuries found an increase in the frequency of scald and contact burn injuries at home, thought to be secondary to the stay-at-home orders imposed for the pandemic.^11^

It has previously been shown that homeless patients on medical and surgical services remain hospitalized longer than housed patients due to a lack of respite care and resources for these patients to recuperate following admission.^1^ This was compounded by the disproportionate effect of the COVID-19 pandemic on the homeless population, as they experienced further barriers to adequate housing as well as healthcare due to a multitude of factors, including shelters lacking the capacity to allow residents to self-isolate, limited staff and resources, limitations in social work and health care services, as well as difficulties navigating COVID-19 regulations.^12^

A prolonged hospital admission further increases the opportunity for hospital-related complications, such as infection.^4^ Thus, LOS has implications both on the individual and community level. To date, no study has characterized the impact of COVID-19 on LOS following acute burn injury or its effects on the homeless community. As suggested by previous studies exploring the impact of COVID-19 on healthcare services, acute burn care would likely have been impacted, given the heavy dependence on resources such as specialized personnel and supplies.

Although burn care is part of the trauma service mandate and was maintained at Vancouver General Hospital (VGH) throughout the COVID-19 pandemic, acute burn care itself was not measurably compromised. Emergent surgeries, resuscitation, and multidisciplinary acute management proceeded without delays, and patients continued to receive timely and complete trauma-level burn care. However, non-acute components of burn services were variably affected by system-wide adaptations to the pandemic. These included redeployment of allied health professionals, interruptions in outpatient rehabilitation and wound care clinics, stricter infection control protocols that altered workflow, and barriers to discharge planning. While these issues did not alter acute trauma care delivery, they did influence the broader continuum of burn care.

The primary objective of this study is to examine the relationship between the LOS in hospital before and during the COVID-19 pandemic at a quaternary provincial burn unit from 2016-2022. April 1, 2020, will be considered the cut-off for the pre-COVID-19 period. The secondary objective will be to compare how lack of housing (defined as the patient having no fixed address) in patients contributes to LOS in hospital.

## METHODS

### Study area and setting

The Vancouver Coastal Health (VCH) region is the most extensive publicly funded healthcare system in British Columbia, Canada, serving diverse ethnic and socioeconomic populations.^13^^,^^14^ Within this system, VGH is the sole facility designated as a Level 1 Burn and Trauma Centre for adults. The Burns, Trauma, and High Acuity Unit (BTHAU) and Burn Clinic are equipped to provide acute and reconstructive care for patients with severe burns.^13^^,^^15^ Patient care is multidisciplinary, including a surgical team, nurses, occupational therapists, physiotherapists, dietitians, music therapist, psychiatry, pain and addiction services and social workers.^15^

### Patient data

This retrospective, population-based study was approved by the University of British Columbia Clinical Research Ethics Board (REB#: H21-00598). Data were derived from the VGH Burn Clinic database to identify all adult patients (age ≥ 18 years) admitted to a quaternary provincial burn unit from April 1, 2016, to March 31, 2022. Patients were divided into two groups: if admitted prior to April 1, 2020, they were considered the pre-COVID cohort; if admitted April 1, 2020, onwards, they were considered the COVID cohort. The end of the COVID-19 study period (March 31, 2023) was selected to encompass the final fiscal year in which pandemic-related operational disruptions were variably present at VGH. While provincial restrictions began lifting in April 2022, institutional operations made a more gradual return. Exclusion criteria included patients under 18 years of age at the time of injury, patients not initially admitted to the burn unit, and records with missing data. Ethnicity was collected descriptively to characterize the patient population and was not used as a surrogate for socioeconomic status or marginalization.

Key supporting variables in the analysis included patient demographics, date of injury (pre/post-COVID), LOS, housing status (with or without a fixed home address), discharge disposition, and total body surface area (TBSA). Discharge disposition included to home with or without supports, shelter/street, jail, transfer to another ward, service, hospital, rehabilitation facility, or self-discharge against medical advice (AMA). Circumstances of injury, inhalational injury, and ethnicity were also assessed. Ethnicity derived from patient charts was categorized as Caucasian, Asian, Hispanic, Indigenous, East Indian, Black, or unknown as defined in the NTRACS burn registry program.

### Statistical analysis

Multivariable logistic regression was used to model associations between the variables described above. Odds ratios, confidence intervals, and *P*-values with significance set at *P <* .05 are reported. Descriptive statistics are presented as mean and standard deviation (normally distributed continuous variables), median and interquartile ranges (non-normally distributed continuous variables), and proportions (categorical data). Univariable comparisons of continuous variables were performed using independent t-tests for normally distributed data and the Wilcoxon rank-sum test for non-normally distributed data. All statistical analyses were performed using the SPSS software package version 27.0 (SPSS Inc., Chicago, IL).

## RESULTS

### Trends and patient characteristics

#### Pre-COVID

Between April 1, 2016, and March 31, 2020, a significant cohort of patients meeting inclusion criteria were admitted to the BTHAU (Table 1). The demographic profile highlighted a predominance of males over females, with Caucasians being the most common ethnic group, followed by Asians and Indigenous peoples, among others. The most frequent cause of injury was fire/flame, with scalds and contact injuries also notable. A large majority of patients had a fixed address. No difference was observed between gender, ethnicity, or type of residence on LOS.

**Table 1:** Comparing Characteristics of Acute Burn Admissions Between the Pre-COVID and COVID-19 Periods

Variable	**Pre-COVID-19**		**COVID-19**		***P*-value**
**Total patients**	301		197		
**Median Age** (years)	47.7		47.1		.671
**TBSA** (%)	12.4%		13.5%		.767
**Length of stay** (days)	20.0		21.5		.034
**Length of stay** (days)/% TBSA	2.9		3.4		.487
**Sex** Female Male	74 227	24.6% 75.4%	48 149	24.4% 75.675.6%	.119 .123
**Ethnicity** Caucasian Asian Indigenous East Indian Hispanic Black Unknown/Other	184 34 26 13 4 2 38	61.1% 11.3% 8.6% 4.3% 1.3% 0.7% 12.6%	106 11 17 8 0 2 53	53.8% 5.6% 8.6% 4.1% 1.0% 26.9%	**<.001** .509 .653 .268 .236 .321
**Etiology** Fire/Flame Scald Contact Electrical Chemical Radiation Unknown/Other	200 47 22 20 5 1 6	66.4% 15.6% 7.3% 6.6% 1.7% 0.3% 2.0%	138 30 16 9 1 1 2	70.1% 15.2% 8.1% 4.6% 0.5% 0.5% 1.0%	.620 .578 .359 .217 .298 .365 .558
**Residence** House/apartment No fixed address Other	244 50 7	81.1% 16.6% 2.3%	144 52 1	73.1% 26.4% 0.5%	**.045** **.008** .543

*P*-values <.05 are boldfaced. Abbreviation: TBSA, total body surface area.

#### COVID

During the COVID period, from April 1, 2020, and March 31, 2023, a diverse group of patients meeting inclusion criteria were admitted to the BTHAU (Table 1). The patient profile remained predominantly male with a slight shift in ethnic composition, where Caucasian patients were still the most common group, but with a considerable portion of patients of unknown ethnicity. Fire/flame injuries continued to be the leading cause of admission, alongside scald and contact injuries. The residence status demonstrated a majority with fixed address, and similar to the pre-COVID period, the LOS showed no disparities based on gender, ethnicity, or residence status.

### COVID-19 impacts

A 1.5-day longer LOS was observed during COVID compared to prior (*P* < .05). No difference was noted in mean TBSA or gender between these two time periods. As for ethnicity, it was found that Caucasian patients had a mean LOS of 31.3 days during COVID compared to 20.7 days pre-COVID (*P* < .05), with no difference in TBSA in this ethnic group between the two time periods.

Further, patients with a defined address code had an 18.9-day LOS pre-COVID compared to 24.0 days during COVID-19 (*P* < .05). Notably, there was a 58.9% increase in the proportion of patients with no fixed address admitted to hospital during COVID (52 of 197 patients) compared to pre-COVID (50 of 301 patients) (*P* < .05).

No differences were noted otherwise in TBSA or residence within individual ethnic or gender groups between the two time periods.

When LOS was stratified by TBSA (Table 2), increased LOS was noted during the COVID time period compared to the pre-COVID time period in the 0%-5% group, with a 3.8-day increase, the 16%-20% group with a 12.8-day increase, the 26%-30% with a 20.0-day increase, and the 36%-40% TBSA group with a 21.1-day increase (*P* < .05).

**Table 2:** Length of Stay by Total Body Surface Area Stratification

**TBSA (%)**	**Mean LOS (days)**		***P*-value**
	**Pre-COVID**	**COVID**	
0%-5%	6.7	10.5	**.00484**
6%-10%	13.8	11.9	.2233
11%-15%	18.0	18.6	.4323
16%-20%	16.0	28.8	**.00410**
21%-25%	36.2	35.2	.4495
26%-30%	32.9	52.9	**.0480**
31%-35%	79.1	39.5	.1665
36%-40%	43.9	65.0	**.0495**
41%-45%	42.0	41.0	.4208
46%-50%	72.0	Data not available	–
51%-55%	74.5	Data not available	–
56%-60%	166	111.5	.3285
>60%	Data not available		–

*P*-values <.05 are boldfaced. Abbreviations: LOS, length of stay; TBSA, total body surface area.

## DISCUSSION

This study investigates the impact of the COVID-19 pandemic on the duration of hospital stay and the demographics of burn injury patients. Such analyses are essential in providing insights into the ever-evolving landscape of burn care during crises, such as a global pandemic. An understanding of these relationships might help inform an approach to acute burn management in such settings. Still, it can be applied to resource-limited settings where acute treatment might be delayed.

A significant difference was noted in LOS between the pre-COVID and COVID-19 periods, with an overall 1.5-day increase in admission during COVID-19. It is important to note that while LOS was used as a primary outcome, acute burn care at VGH remained consistent during the pandemic. Emergent surgical interventions, resuscitation, and inpatient multidisciplinary care were delivered without measurable compromise in quality, timeliness, or completeness. The observed differences in LOS likely reflect delays related to non-acute aspects of care, including access to rehabilitation services, outpatient follow-up, discharge planning, and step-down resources, all of which were variably constrained by broader institutional adaptations to COVID-19. Thus, while the acute trauma mandate for burns remained intact, ancillary and post-acute services were disproportionately affected.

It is important to note that while LOS was used as a primary outcome, we were unable to distinguish between patients who remained in hospital due to ongoing medical needs versus those whose discharge was delayed for non-clinical reasons. Readiness-for-discharge was not consistently recorded in the VGH burn registry or inpatient notes. However, anecdotally, many patients during the COVID-19 period remained admitted beyond medical necessity due to placement delays, limited community resources, and pandemic-related discharge criteria. This highlights the importance of interpreting LOS as a composite measure reflecting both clinical recovery and systemic discharge capacity.

When LOS was stratified by TBSA groups, much greater differences were noted in LOS dependent on TBSA group. The overall 1.5-day increase in LOS can be attributed to numerous factors, such as the severity of injury necessitating a more extended hospital stay; however, the mean TBSA between the two periods was grossly similar.^16^ When stratified by TBSA, greater differences were noted. This can be explained as with the onset of the pandemic, hospital protocols, including resource allocation and the ability to provide specialized services, including occupational therapy, physiotherapy, and social work, may have contributed to prolonged stays.^16^^,^^17^ This could have been compounded by reduced availability of post-discharge care, such as outpatient and in-home services, such as wound care.^16^^,^^18^

Increased LOS places emotional and psychological stress not only on patients but also on their supports as well, potentially impacting overall recovery and well-being.^19^ On a systemic level, longer stays can place a significant strain on healthcare resources, leading to higher medical costs and decreased resources such as beds, impacting the ability to provide timely care for other patients.^6^^,^^20^

A notable increase was noted in LOS for Caucasian patients, who stayed 31.3 days during COVID-19, compared to 20.7 days pre-COVID. This increase may be attributed to socio-economic factors influencing access to care or indicate a variation in the severity or nature of injuries sustained by this demographic. This aspect warrants further investigation to elucidate specific causes for this increased LOS and ensure equitable care for all ethnic demographics.

Our study also demonstrates a significant rise in the number of homeless patients admitted with burn injuries during the pandemic. The 58% increase in patients with no fixed address raises an alarming trend, underscoring the worsening of homelessness during the pandemic. It highlights the vulnerability of people experiencing homelessness during crises, with limited access to safe shelter and healthcare. This rise in homelessness can be attributed to economic downturns, job losses, and disruptions in social services contributed by the COVID-19 pandemic, further exacerbated by pandemic-related resource limitations and restrictions.^12^^,^^21-23^ It is well known that homelessness is a significant risk factor impacting health outcomes, including acute burn injuries.^24^ Such individuals, often lacking access to safe living conditions, are at increased risk of injury, including burns.^25^ For example, inadequate shelter may lead to unsafe heating methods to stay warm, such as lighting a fire in an outdoor tent and reliance on hazardous cooking methods in improvised shelters.^25^ There is a need for targeted interventions and support systems to mitigate risks and provide adequate care for these individuals. A re-evaluation of already-existing support systems is also required to better mitigate risk and allocate resources for homeless individuals during extraordinary circumstances like a global pandemic.

Interestingly, while the proportion of unhoused patients increased significantly during the COVID-19 period, their median LOS remained stable. This may reflect a “ceiling effect,” wherein baseline discharge barriers—including limited access to respite care, shelter placement, or outpatient support—already contributed to prolonged admissions pre-COVID. The pandemic strained these systems further but may not have exacerbated delays in a quantifiable way for this group. In contrast, housed patients who would typically have timely discharge pathways experienced new challenges, including limited access to rehabilitation, suspended outpatient services, and logistical delays related to isolation protocols. These shifting dynamics may explain the overall increase in LOS observed during the COVID-19 period despite no change in LOS among the unhoused subgroup.

While no difference was found in hospital LOS between the two time periods in patients with no address, the contrary was noted in individuals with fixed addresses, with 18.9-day LOS pre-COVID compared to 24.0 days during COVID. Possible reasons could include injury severity, changes in hospital protocols resulting in longer observation or isolation periods, concomitant COVID-19 infections, or limited inpatient and outpatient resources such as wound care or home care support; however, our data does not fully support this. While only two patients in our COVID-19 cohort had concomitant COVID-19 infections during admission, both experienced prolonged hospitalizations. These patients were managed under isolation protocols, which may have impacted the timing of assessments, dressing changes, or discharge logistics. However, due to the small sample size and confounding burn severity, no conclusions can be drawn regarding the independent impact of COVID-19 positivity on LOS. In our sample, injury severity (TBSA) was, on average, similar across both time periods, and there were no overt changes in hospital protocols in terms of burn management. Furthermore, only two patients were identified in the COVID-19 time period with concomitant COVID-19 infections. Further investigation into other factors, such as delays in discharge planning or resource availability, may provide a more accurate explanation.

The findings of this study lay a foundation for future investigation into understanding the long-term impacts of the COVID-19 pandemic on burn care and patient outcomes, but also serve to emphasize the importance of adaptation of such services to address the needs of more vulnerable populations who are often disproportionately affected during such events. Responsive healthcare strategies are essential to address the evolving needs of these populations.

This study had multiple limitations, primarily its retrospective nature, as data were collected from pre-existing records, which may have inconsistent data quality or incomplete information. For example, the dataset categorized a significant portion of patient ethnicities as “Unknown/Other.” This categorization introduces ambiguity, as it prevents a thorough understanding of the ethnic distribution of the study population and could potentially bias interpretations of demographic impacts on burn injury patterns and outcomes. An inherent limitation of our study design is the unequal duration of the pre-COVID (4 years) and COVID (3 years) cohorts. While this may affect comparisons of aggregate admission volumes or trends, our primary outcome—LOS—is an individual-level variable analyzed across independent samples. Therefore, the difference in cohort length does not compromise the statistical validity of our comparisons. Future studies might consider balanced cohorts or time-matched designs if incidence data is the focus. Further, the absence of a well-defined point marking a total return to routine healthcare services post-COVID-19 could mean that the study’s timeframe might inadvertently include some patients whose care was not truly influenced by pandemic-related changes. This factor adds a layer of complexity in interpreting the data. It might slightly skew the results, particularly regarding the length of hospital stays and the nature of burn injuries treated during this transitional period. Additionally, in hospital, morbidities such as infection and graft failure were not consistently documented and could not be reliably included in the analysis. This limits our ability to quantify how these clinical complications may have contributed to variations in LOS between cohorts.

## CONCLUSION

This study demonstrates the impacts of COVID-19 on the LOS in hospital following an acute burn injury, underscoring the complex interplay between global crises and individual burn injury outcomes. While a 1.5-day increase in LOS was observed, what was more highlighted was the significant increase in patients with no fixed address admitted to hospital following injury, emphasizing the need for adaptable healthcare strategies and targeted interventions for at-risk populations, especially during unprecedented events like the COVID-19 pandemic.

## Statement of Ethics

This single-center, retrospective cohort study was approved by the University of British Columbia Clinical Research Ethics Board (REB#: H22-03595).

## Notes

Author contributions: Sara Sheikh-Oleslami (Conceptualization [equal], Data curation [equal], Formal analysis [lead], Investigation [lead], Methodology [equal], Software [equal], Visualization [lead], Writing—original draft [lead], Writing—review & editing [equal], involved in developing the study protocol, involved in data collection, involved in analysis and manuscript writing in consultation with AP), Bettina Papp (Data curation [equal], Writing—review & editing [equal], involved in data collection, involved in the critical revision of the manuscript, with AP providing final approval of the manuscript for submission), and Anthony Papp (Conceptualization [lead], Methodology [equal], Project administration [lead], Resources [lead], Supervision [lead], Writing—review & editing [lead], involved in developing the study protocol, involved in the critical revision of the manuscript, with AP providing final approval of the manuscript for submission)
